# Sperm Preparation with Microfluidic Sperm Sorting Chip May Improve Intracytoplasmic Sperm Injection Outcomes Compared to Density Gradient Centrifugation

**DOI:** 10.1007/s43032-024-01483-1

**Published:** 2024-02-23

**Authors:** Maria Banti, Estee Van Zyl, Dimitrios Kafetzis

**Affiliations:** Orchid Reproductive & Andrology Services, Dubai Healthcare City, Unit 4016, Block E, Al Razi Bldg #64, Dubai, UAE

**Keywords:** ICSI, Microfluidic sperm sorting (MSS), Density gradient centrifugation (DGC), Microfluidic device, Euploidy, Sperm preparation

## Abstract

Does sperm preparation using the FERTILE PLUS™ Sperm Sorting Chip improve fertilization rates, blastocyst formation, utilization, and euploidy rates in patients undergoing intracytoplasmic sperm injection (ICSI), compared with density gradient centrifugation (DGC)? A single-cohort, retrospective data review including data from 53 couples who underwent ICSI cycles within a 12-month period. For each couple, the two closest, consecutive cycles were identified, where one used the standard technique of sperm preparation (DGC) and the subsequent used FERTILE PLUS™, therefore, couples acted as their own controls. Paired samples *t*-test was used to compare means for the outcomes (fertilization, blastocyst formation, utilization, and euploidy rates). Binary logistic regression analysis assessed the relationship between female age, the presence of male factor infertility, and euploidy rates. Blastocyst, utilization, and euploidy rates were significantly higher for cycles using FERTILE PLUS™ compared to DGC (76% vs 56%, *p* = 0.002; 60% vs 41%, *p* = 0.005, and 40% vs 20%, *p* = 0.001, respectively). Although there was an increase in fertilization rates for cycles using FERTILE PLUS™, this was not significant (72% vs 68%, *p* = 0.449). The euploidy rates of females ≤ 35 years were significantly increased when the FERTILE PLUS™ sperm preparation method was used, compared to the older age group (OR 2.31, *p* = 0.007). No significant association was found between the presence or absence of male factor infertility and euploidy rates between the two cycles. This study provides tentative evidence that the FERTILE PLUS™ microfluidic sorting device for sperm selection can improve blastocyst formation, utilization, and euploidy rates following ICSI in comparison to the DGC method.

## Introduction

Infertility, defined by the failure to conceive after at least 12 months of regular unprotected sexual intercourse, is estimated to affect around 17.5% of the adult population [1] or 8–12% of reproductive-age couples worldwide [2]. The etiology of infertility is multifactorial and complex and may involve factors relating to the male or female reproductive systems or a combination of both [3]. Infertility can significantly impact psychosocial well-being, prompting numerous couples to seek infertility treatment. Assisted reproductive technology (ART) has advanced considerably since the birth of Louis Brown—the first baby conceived through in vitro fertilization (IVF) in 1978 [4]. The utilization of ART is increasing as the effectiveness and safety of these technologies have improved over the years. However, there is still room for improvement in achieving higher success rates with fewer cycles [4].

Sperm selection is a vital part of all ART procedures, as sperm quality influences success rates and health in offspring [5]. However, since the start of ART, there has been little technological innovation for sperm preparation methods until the recent years [6–8]. During natural conception, sperm undergo a complex natural selection process, involving multiple stages during their long journey through the female reproductive tract, so that only the more motile, higher quality sperm reach the oocyte with the potential for successful fertilization. Only 1000 or less of the millions of sperm contained in a single ejaculate reach the fallopian tube, and only a small proportion of these reach the ampulla, or site of fertilization [9–11]. ART bypass stages of the natural sperm selection process in various ways. To avoid fertilization with a defective sperm, semen preparation techniques aim to select high-quality sperm by attempting to mimic aspects of the in vivo sperm selection process [10].

In conventional IVF, sperm and oocytes are mixed in a petri dish and left for fertilization to occur. Intracytoplasmic sperm injection (ICSI) is a technique in which a single sperm is injected into a mature oocyte, thereby bypassing the final barriers of sperm-oocyte interaction that the spermatozoa must overcome to achieve fertilization [12]. ICSI is the method of choice for male factor infertility, but has also been shown to be effective in other circumstances, such as unexplained infertility [13]. ICSI has become a more commonly used method of fertilization over conventional IVF in many areas of the world, including the United Arab Emirates, due to the technique’s consistency and versatility [14, 15]. However, sperm selection for ICSI is even more crucial than for other ART, in order to maximize the chance of fertilization with an optimal spermatozoon [14, 16].

Conventional methods of semen preparation include sperm washing, swim up, and density gradient centrifugation (DGC). DGC, the current standard sperm preparation method for IVF, utilizes differences in the density to select the best sperm, followed by washing using centrifugation [17]. However, DGC is time and labor intensive, with multiple operator-sensitive steps. In addition, centrifugation has been shown to increase reactive oxygen species (ROS) formation that may induce sperm DNA fragmentation (sDF) which could negatively impact sperm function and therefore fertilization and embryo development [8, 18, 19].

Advanced sperm preparation approaches have been developed to mimic the physiological selection that occurs in the female genital tract, simplify semen preparation, and avoid the use of centrifugation. Some sperm selection techniques include methods based on sperm membrane markers, such as hyaluronan or annexin [7]. The application of microfluidic-based technologies for sperm preparation and selection is a rapidly developing area in ART. Microfluidics is the science and technology of accurate manipulation of small amounts of fluids [20]. In microfluidic sorting devices, sperm typically travel through micro-channels whose dimensions hydrodynamically constrain the migration of compromised sperm while allowing motile sperm to progress to the outlet [21]. Microfluidic, motility-based sperm selection methods attempt to mimic the natural progression of sperm through the female reproductive tract, including physical aspects of the fallopian tubes [8, 10]. Microfluidic sorting selects sperm according to size, motility, and other characteristics, such as DNA integrity, without the need for centrifugation [18, 21, 22]. Microfluidic-based sperm separation (MSS) devices are proposed as a simple, reliable, and standardized method to improve ART outcomes, by mitigating against the production of reactive oxygen species and selecting high-quality, motile sperm [7, 10, 19, 23–25].

The FERTILE PLUS™ method is a standardized method with an easy-to-follow protocol that is far less dependent on the skill or experience of the embryologist than other methods, such as DGC. The FERTILE PLUS™ (850 µL) Sperm Sorting Chip is a single-use, flow-free, dual chambered, microfluidic-based sperm sorting device. FERTILE PLUS™ was previously known as Zymot, prior to a name change by the manufacturer. The lower chamber contains a sample inlet and fluid channel separated from the upper collection chamber by a microporous membrane with 8-μm pores, demonstrated as the optimal size for selection of sperm with higher motility and normal morphology [22, 26]. The design of the sperm sorting chip utilizes sperm forward motility to sort healthy motile sperm from compromised, poorly motile sperm present in the raw semen sample. After sperm enter the lower channel through the inlet, the more motile sperm swim through the micro-channel and up through the filter pores within the membrane to reach the outlet. The FERTILE PLUS™ (850µL) Sperm Sorting Chip produces a 500 µL sample that can be used for ICSI, IVF, or intrauterine insemination (IUI). This easy-to-use method with fewer sample manipulation steps offers increased reliability and significant time saving over traditional sperm preparation techniques. However, as microfluidic sorting produces lower sperm concentration yields than other techniques and sperm recovery is highly dependent on the quality of the semen sample, sperm concentration must be at least 10 million per milliliter for optimal use of FERTILE PLUS™.

Evaluating outcomes of MSS devices compared to other sperm preparation methods for ART is a relatively new area of research which has, so far, shown conflicting results [27, 28].

## Aim of the Study

The aim of this study is to investigate whether sperm preparation using the FERTILE PLUS™ Sperm Sorting Chip improves fertilization, blastocyst, utilization, and euploidy rates compared to DGC sperm preparation in patients undergoing ICSI.

## Materials and Methods

This is a single-cohort, single-centered retrospective data review to compare the effect of two different sperm preparation methods on outcomes from ICSI cycles. The clinic used the standard protocol for DGC prior to February 2021 and started using the FERTILE PLUS™ for all couples (except those with very low sperm count) from February 2021 onwards. Records were accessed for all clinic patients who underwent at least 2 cycles at Orchid Fertility Center (Dubai, UAE) within a 12-month period overlapping February 2021. Data was gathered on the age of the female and indication for ART. Sperm morphology was also assessed to be either normal or below normal range, according to the WHO 5th edition criteria (2010) [29]. Samples with 4% normal morphology or above were classified as normal.

For each couple, the two closest cycles were identified, where one used the standard technique of DGC and the subsequent cycle used FERTILE PLUS™ for sperm preparation. Other than the method of sperm preparation, standard treatment protocols were similar between cycles. In this way, couples acted as their own controls for comparison of the outcomes of the different sperm selection techniques. The outcomes analyzed were fertilization, blastocyst and utilization rates. Euploidy rates were also recorded for couples who opted for Preimplantation Genetic Testing for Aneuploidy (PGT-A) to identify euploid embryos.

Ethical approval was obtained from the Dubai Scientific Research Ethics Committee Review Board (reference number: DSREC-02/2023_15). The study was exempted from the requirement of informed consent as all data was collected retrospectively and anonymously, with no link to personal identifiers, and treatment was in no way modified for the purpose of the study.

### Inclusion Criteria

This study included the two closest cycles of couples who underwent ICSI at the clinic within a 12-month period, where the one cycle used DGC and the subsequent used FERTILE PLUS™.

### Exclusion Criteria

This study excluded males with sperm samples with less than 10 million per milliliter concentrations and females with a BMI higher than 30.

## Clinical and Laboratory Procedures

### Ovarian Stimulation

For each ART cycle, ovarian stimulation was commenced on the 2nd or 3rd day of menstruation as per globally established standards for an antagonist protocol, following an ultrasound evaluation of follicular count and exclusion of ovarian cysts. A comprehensive hormonal assessment, including FSH, LH, AMH, estradiol (E2), and progesterone, along with BHCG, was conducted. The choice of gonadotrophin dose and type (recombinant vs urinary) was determined based on these evaluations, considering any previous cycles and the ovarian response in those cycles.

After 10–12 days of ovarian stimulation in an autologous protocol, with ongoing assessments of follicular size, growth, and hormonal levels (E2 and LH), oocyte retrieval took place. The retrieval occurred 36 h post-HCG or GnRH administration. The serum E2 levels at the trigger phase ranged between 500 and 1000 pmol/L per follicle. To mitigate the risk of hyperstimulation, patients with E2 levels exceeding 10,000 pmol/L were triggered only with agonist trigger (triptorelyn 0.2 or 0.3 mg). All mature oocytes retrieved were injected using ICSI.

### Sperm Preparation

The raw semen was allowed to liquefy for 20–30 min before preparation, following a controlled period of sexual abstinence lasting between 2 and 7 days, synchronized with the timing of oocyte retrieval.

1. DGC Sperm Preparation Method
  Silane-coated silica particles were used for gradient preparation (PureCeption™, CooperSurgical, Denmark). PureCeption 100% was diluted to aliquots of 90 and 45% using sperm washing media (Quinn’s™ Sperm Washing Medium, CooperSurgical, Denmark). A two-layered gradient was prepared after media reached room temperature. Using a sterile pipette, up to 2 mL of liquefied sample was overlaid into the conical 12-mL centrifuge tube. The tube was centrifuged at 300 g for 20 min. The pellet was then transferred with a sterile Pasteur pipette to a new conical tube in a volume of 3 mL pre-equilibrated at 37°C sperm washing medium and again centrifuged at 500g for 10’. The pellet was then moved to a sterile 5-ml round bottom tube and re-suspended to 0.5–1 mL of fertilization media (Quinn’s Advantage™ Protein Plus Fertilization media, CooperSurgical, Denmark). An aliquot was examined for sperm concentration and motility, and the sample was incubated at 37°C and 6% CO2 until the time of ICSI.
2. FERTILE PLUS™ Method of Sperm Preparation
  Eight hundred fifty microliters of the semen sample was slowly drawn up with a syringe and injected into the inlet of a FERTILE PLUS™ Microfluidic Sperm Sorting Chip. Seven hundred fifty microliters of pre-equilibrated at 37°C sperm washing medium (Quinn’sTM Sperm Washing Medium, CooperSurgical, Denmark) was drawn up a fresh syringe and added to the upper collection chamber in order to cover the membrane surface. The FERTILE PLUS™ Microfluidic Sperm Sorting Chip was incubated at 37°C for 30 min. Following this, 500 μL of processed sample was slowly aspirated with a syringe from the outlet and transferred to a sterile 5 ml round bottom tube. An aliquot was examined for sperm concentration and motility, and the sample was incubated at 37°C in a non CO^2^ incubator until the time of ICSI.

### ICSI Procedure

For the ICSI procedure, cumulus and corona radiata cells were removed 2 h post oocyte-retrieval, by aspiration of the oocytes through 150-mm glass Pasteur pipette in Hyaluronidase 80 U/mL (Hyaluronidase, CooperSurgical, Denmark) and washing of the oocytes in HEPES-buffered medium (Quinn’s Advantage™ Medium with HEPES, CooperSurgical, Denmark). Only metaphase II oocytes were injected. ICSI was performed 39–40 h post trigger injection in HEPES-buffered medium (Quinn’s Advantage™ Medium with HEPES, CooperSurgical, Denmark) on a heated microscope stage at 37°C. For the injection, a spiked tip ICSI Injection Pipette with a 30° angle (ICSI injection Pipettes, Sunlight Medial. Inc., Florida, USA) and a holding pipette with a 30° angle (Holding Pipettes, Sunlight Medial. Inc., Florida, USA) were used. The sperm was observed under the inverted microscope at high magnification (× 20), and normal looking, motile sperm was chosen to be injected into the eggs. No additional sperm selection methods such as PICSI or IMSI were employed. Sperm were immobilized in polyvinylpyrrolidone (PVP, CooperSurgical, Denmark) by breaking the tail with the shaft of the injection pipette. Using gentle suction, oocytes were positioned using a holding pipette with the polar body at 6 o’clock or 12 o’clock, while immobilized sperm were moved to the injection pipette’s tip and inserted through the zona at 3 o’clock to puncture the oolemma, as indicated by a sudden ooplasm shift, before sperm disposition. The oocytes were transferred immediately after injection in single step medium (SAGE 1-Step™, CooperSurgical, Denmark) for uninterrupted embryo culture and cultured in the EmbryoScope Time-lapse incubator (EmbryoScope time-lapse system, Vitrolife, Sweden).

### Embryo Assessment

Normal fertilization was confirmed by the presence of two pronuclei (2PN) and two polar bodies. Normally fertilized zygotes were cultured until day 5 or 6 in uninterrupted culture in single step medium (SAGE 1-Step™, CooperSurgical, Denmark) in the EmbryoScope Plus Time-lapse incubator (EmbryoScope time-lapse system, Vitrolife, Sweden). On day 5 and day 6, blastocyst formation was assessed, and the number of suitable quality blastocysts was recorded (see Appendix for Blastocyst Grading Criteria). Blastocysts graded 3BC and above were considered suitable for utilization. These embryos were then used for fresh transfer or freeze-all, based on treatment plan. The majority of couples (94.3%) opted for PGT-A. Fertilization, blastocyst, and utilization rates were assessed. Euploidy rates were also analyzed for couples who underwent PGT-A.

## Data Analysis

Statistical analysis was performed with Statistical Package for Social Sciences (SPSS v.28, IBM Corp.). Descriptive statistics for the study population included percentage distributions for female age and indications for ART. Outcome variables (fertilization, blastocyst, utilization, and euploidy rates) were analyzed for each individual couple for the different cycles using sperm preparation with DGC or FERTILE PLUS™, and the mean values ± standard error of the mean (SEM) were reported. Similar methods of data analysis, where couples acted as their own controls to compare cycle outcomes, were employed in previous studies [30, 31]. Paired samples *t*-test was used to compare means for the outcomes between the DGC and FERTILE PLUS™ sperm preparation methods. By performing a *t*-test the individual differences before and after the procedure are assessed instead of analyzing the couples as a group, which would lead to the equal distribution of the risk factors and would not be scientifically accurate. This eliminates individual risk factors that can affect the results of the different sperm preparation methods.

Data was further stratified by female age group (≤ 35 years or > 35 years) and the presence or absence of male factor infertility. Odds ratios, 95% confidence intervals, and *p*-values were calculated for increased euploidy rates following the use of FERTILE PLUS™. Finally, binary logistic regression analysis was employed to assess the relationship between female age (≤ 35 years or > 35 years) and the presence or absence of male factor infertility with increased euploidy rates.

## Results

### General Characteristics and Indications for ART of the Study Population

Data of 53 couples were included in this study. The mean female age was 37.2 years (SD 3.7 years). The majority of females (75.5%) were aged 35 years and above, and the majority of couples (81.2%) were recorded as having male factor infertility (Table 1). The indications for ART for the 3 remaining couples were unexplained infertility and family balancing. Out of the 53 couples, 49 (92.45%) had sperm morphology below the normal range, and 40 couples (75.5%) were assessed to be poor prognosis due to reasons including multiple previous failures and advanced maternal range in combination with severe male factor. Out of the 53 couples, 50 (94.3%) underwent PGT-A.

**Table 1:** General characteristic of the study population

Characteristics (*n*, 53)	*N*	%
Female age group (years)		
< 35	13	24.5%
35–37	16	30.2%
38–39	10	18.9%
40–42	9	17.0%
> 42	5	9.4%
Indications for ART		
Age only	7	13.2%
Male factor only	18	34.0%
Age and male factor	25	47.2%
Others	3	5.7%

### Outcomes of Different Sperm Preparation Methods

**Fig. 1:**
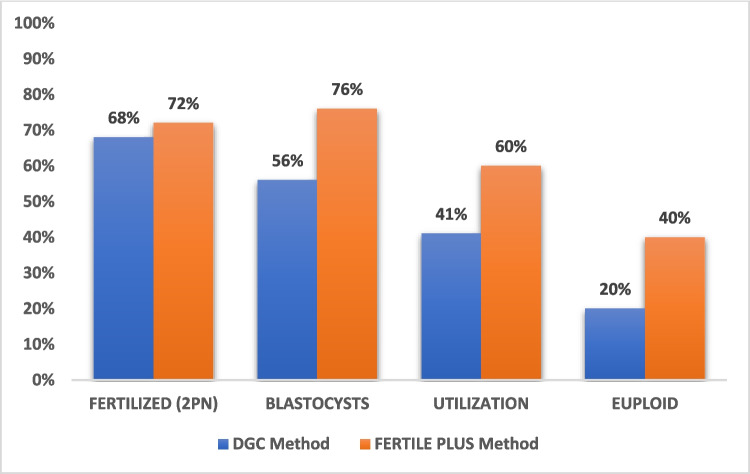
Mean outcome rates between the different sperm preparation methods for all couples

The results of the two different sperm preparation methods were analyzed using paired *t*-test (Table 2). The total number of oocytes collected was exactly the same (*n* = 506) for the two groups, while the number of mature oocytes injected were higher in the FERTILE PLUS™ group (*n* = 406 vs *n* = 368). The number of blastocysts biopsied was 84/97 (86.6%) for the DGC cycles and 133/159 (83.6%) for the FERTILE PLUS™ cycles. There was a trend for higher fertilization rate in the FERTILE PLUS™ group (72% vs 68%); however, this was not statistically significant (*p* = 0.449). Blastocyst, utilization, and euploidy rates were significantly higher for cycles using FERTILE PLUS™ compared to DGC (76% vs 56%, *p* = 0.002; 60% vs 41%, *p* = 0.005; and 40% vs 20%, *p* = 0.001, respectively). Analysis of different subgroups was not performed due to the low number of patients in each subgroup, which would not lead to meaningful conclusions. The outcomes are further illustrated in Fig. 1.

**Table 2:** Differences in outcomes based on sperm preparation method

	**DGC Method**		**FERTILE PLUS™ Method**		**Total**
No oocytes collected	506		506		1012
No mature oocytes	368		406		774
Outcomes	***n***	**mean ± SEM**	***n***	**mean ± SEM**	***p*-value**
Fertilized (2PN)	254	0.68 ± 0.036	289	0.72 ± 0.026	0.449
Blastocysts	143	0.56 ± 0.049	212	0.76 ± 0.035	0.002*
Utilization	97	0.41 ± 0.047	159	0.60 ± 0.038	0.005*
Euploid	28	0.20 ± 0.044	58	0.40 ± 0.050	0.001*

*Paired *t*-test. *p*-value < 0.05

### Association of Infertility Indication with Euploidy Outcomes

Table 3 shows odds ratios for increased euploidy rates following the use of FERTILE PLUS™ compared to DGC based on female age group (≤ 35 years or > 35 years) and the presence or absence of male factor infertility. In the younger age group (≤ 35 years), the odds of attaining increased euploidy rates was 2.3 times higher (95% CI, 1.34–3.99, *p* = 0.007) compared to the odds of attaining increased euploidy rates in the older age group (≤ 35 years). Male factor did not show any significant association with the increased euploidy rates.

**Table 3:** Association of age and male factor infertility with euploidy outcome post FERTILEPLUS

Factors		Total (n)	Increased euploidy%	Odds ratio (95% confidence interval)	*p*-value
Age group	≤ 35 years	16	12 (75.0%)	2.31 (1.34–3.99)	0.007*
	> 35 years	37	12 (32.4%)		
Male factor	Yes	43	17 (39.5%)	1.77 (1.02–3.06)	0.156
	No	10	7 (70.0%)		

*p-value < 0.005

The results of binary logistic regression analysis, to assess the relationship between female age (≤ 35 years or > 35 years) and the presence of male factor infertility and increased euploidy rates, are shown in Table 4. A statistically significant association with a sevenfold higher likelihood of obtaining an increased euploidy rate with FERTILE PLUS™ was found for females ≤ 35 years old compared to older females (OR: 7.09, 95% CI, 1.79–28.09, *p* = 0.005). Male factor infertility showed a negative association with euploidy rates (OR: 0.23, 95% CI, 0.05–1.11).

**Table 4:** Binary logistic regression analysis

Factors	B	S.E	Exp (B)	95% CI for Exp (B)	*p*-value
Age	1.958	0.70	7.09	1.79, 28.09	0.005
Male factor	1.491	0.82	0.23	0.05, 1.11	0.067

## Discussion

This study presents compelling evidence that the application of a microfluidic sperm sorting device, FERTILE PLUS™, enhances various facets of sperm selection for ICSI in contrast to the DGC method. Our results demonstrate statistically significant improvements in blastocyst, utilization, and euploidy rates when using FERTILE PLUS™, compared to the DGC method. Nonetheless, this study did not demonstrate a significant increase in fertilization rates.

Previous studies comparing the DGC method of sperm preparation with microfluidic sperm sorting chips for IVF or ICSI cycles have shown conflicting results. Only a limited number of these studies have reported clinical outcomes [32]. In a study involving 57 couples undergoing ICSI cycles, Kocur et al. (2023) found a significantly higher fertilization rate for FERTILE PLUS™: 76.2% compared to 68.8% in the DGC group (*p* < 0.001). The percentage of euploid embryos was also significantly higher (25.3% vs 42.9%, *p* < 0.001) [33]. A retrospective cohort study involving couples undergoing ICSI cycles using DGC and FERTILE PLUS™ by Robles et al. (2021) found improved blastocyst rates (40.2% vs 29.2%, *p* = 0.02) and higher euploidy rates (43% vs 33%, *p* = 0.016) for the FERTILE PLUS™ group [31]. Yildiz et al. (2019) found no statistically significant difference in fertilization and pregnancy rates between IVF cycles utilizing sperm preparation using DGC (*n* = 312) and MSS devices (*n* = 116), in first-time IVF treatment patients. However, in recurrent IVF failure patients, there was a significant difference in fertilization rates but no statistically significant difference in pregnancy rates [34]. Keskin et al. (2022) investigated whether utilizing MSS improves embryo quality and euploidy rates compared to DGC in couples with repeated implantation failure (RIF) and high SDF. They found blastocyst formation was significantly increased (*p* < 0.001), but not other measures (fertilization rates, euploidy rates, live births) [35]. A study by Anderson et al. (2020) found significantly increased euploidy rates for day 5 embryos (*n* = 358) for FERTILE PLUS™ sperm preparation (63%) compared to DGC (56%; *p* < 0.05) but no significant difference in pregnancy outcomes. Yalcinkaya Kalyan et al. (2022) found no statistically significant differences in laboratory or clinical outcomes between swim-up and FERTILE PLUS™ [36].

Although our study did not directly measure sperm quality parameters following sperm preparation, microfluidic devices are designed to preferentially select highly motile sperm [22, 26]. Previous research has highlighted improvements in morphology, DNA integrity, and motility with microfluidic sperm selection. For instance, Mirsanei et al. (2022) observed significantly higher normal morphology, total motility, and significantly reduced DNA fragmentation in sperm sorted using microfluidic methods. They also reported an increase in fertilization rate and improvement of embryo quality when using microfluidic sperm sorting [28]. Quinn et al. (2018) reported reduced DNA fragmentation in samples processed by microfluidic chips (median DFI = 0%, IQR: 0–2.4) compared to paired samples processed by density-gradient centrifugation with swim-up (median DFI = 6%, IQR: 3–11.5) [21]. Similarly, Pujol et al. (2022) found that utilization of a microfluidic sperm sorting device significantly reduced double-stranded DNA fragmentation by 46% compared to the swim-up method (*p* < 0.001) [37].

An important observation was that the aneuploidy rates in the density gradient group were observed to be slightly lower than the rates in the general population. In previous studies, the average euploidy rate in infertile couples was found to be 33.7% [38]. The observed discrepancy can be attributed to several factors. Firstly, the study population includes a diverse range of infertility causes, including multiple previous failures, male factor infertility combined with advanced maternal age, with 75.5% of the couples considered to be of poor prognosis. The expected euploidy rate in the general population is typically around 40%, and the observed rate with DGC is 20% lower than this anticipated norm. This most likely aligns with the characteristics of the study cohort. Although the results differ from the aforementioned study, the limitations of this study should be emphasized, particularly the smaller sample size and embryo numbers compared to the referenced research.

In conclusion, this study contributes valuable insights into the potential advantages of FERTILE PLUS™ microfluidic sperm sorting over the traditional DGC method in ICSI cycles. While improvements in various parameters were observed, further research is required to comprehensively understand the impact of microfluidic selection on sperm quality and clinical outcomes.

## Strengths and Limitations of Study

This study adds to the overall body of scientific evidence for microfluidic sperm sorting methods. The couples included in this study acted as their own controls, in an attempt to remove bias. However, the findings should be interpreted considering the following limitations.

The main limitation of this study is that a much larger sample size would be required to produce higher powered results. The study design focused on couples returning for a second cycle during the 1-year period in which the new sperm preparation method was introduced. Due to this, achieving a larger sample size during the study period was not feasible. A post hoc sample size calculation (see Appendix) calculated that a sample size of 769 couples would be required to detect a significant difference at 95% CI and with a power of 80%.

Although there were no challenges encountered in identifying the two closest cycles for each couple, returning patients with sperm concentrations less than 10 million per ml did not fit the criteria for the use of FERTILE PLUS™ and therefore were not included in this study. This could add a potential bias.

It is crucial to acknowledge the challenges in achieving complete control over ensuring the consistent oocyte quality between the two cycles compared for each couple, which is a limitation of the study. In order to reduce the bias, the study design included patients returning for treatment within a 1-year period. This timeframe was chosen under the assumption that, within less than 12 months, oocyte quality remains fairly similar. Ensuring a consistent quality of oocytes even within a short period poses inherent difficulties. Various factors can influence oocyte quality, including but not limited to ovarian stimulation, environmental exposures, and lifestyle choices. Even in meticulous efforts to replicate identical stimulation protocols, there exists a degree of unpredictability in the outcome due to the complex nature of these influencing factors. In essence, even with identical ovarian stimulation protocols, there is no guarantee for consistent oocyte quality.

This study did not follow cycle outcomes post embryo transfer. Although the findings of this study suggest an improvement in blastocyst, utilization, and euploidy rates, no information was available on final pregnancy and delivery outcomes at the time of the study. Therefore, it cannot be concluded that outcomes post transfer will be increased.

## Recommendations for Further Research

More large, multi-center, prospective studies using microfluidic sperm selection devices are required to investigate the outcomes and improve the quality and generalizability of research evidence. In order to omit confounding factors, a prospective study would be required to evaluate the two methods of sperm preparation within the same cycle. This could be achieved by dividing the sibling oocytes into two groups and then injecting half of the oocytes with the sperm prepared by DGC and the other half with sperm prepared by the FERTILE PLUS™.

More research is required into potential risks and benefits of microfluidic sperm preparation [39]. Randomized, controlled, clinical trials would provide the highest level of evidence, but the ethical implications of these types of studies require careful consideration. Future studies should investigate the role of microfluidic sperm selection in subgroups, such as those with male factor infertility, previous poor IVF outcomes, or known elevated sperm DNA fragmentation [32].

Studies should also follow post-transfer outcomes on implantation, pregnancies, and resulting offspring. A longer follow-up to record pregnancy and post-birth outcomes would be beneficial, as the quality of sperm has effects beyond fertilization into adult health [10], such as the potential for birth defects and infertility in male offspring [5].

## Conclusion

This retrospective data review provides tentative evidence for improved sperm selection for ICSI cycles using a FERTILE PLUS™, a microfluidic sperm sorting device. However, this is a topic of ongoing research for which larger, prospective trials are required.

## Data Availability

Data and material are available upon request.

## Appendix

### Blastocyst Grading Criteria

Blastocysts graded 3BC and above were considered suitable for utilization.

### Post-hoc Sample Size Calculation

The sample size was calculated using the following formula:

$$n={({Z}_{\alpha /2}+{Z}_{\beta })}^{2}*({p}_{1}(1-{p}_{1})+{p}_{2}(1-{p}_{2}))/{({p}_{1}-{p}_{2})}^{2}$$

where Z_α/2_ is the critical value of the normal distribution at α/2 (e.g., for a confidence level of 95%, *α* is 0.05, and the critical value is 1.96), *Z*_β_ is the critical value of the normal distribution at β (e.g., for a power of 80%, *β* is 0.2, and the critical value is 0.84), and *p*_1_ and *p*_2_ are the expected sample proportions of the two groups.

Based on a previously published study [6], the proportion of expected euploidy from ZYMOT prepared sperm group was considered as 63% (P_1_), and the proportion of euploidy expected from the classic gradient prepared sperm method was considered as 56% (P_2_). Using the above formula, the sample size was calculated to be 769 to detect a significant difference at 95% CI and with a power of 80%. [40]
